# Selection Strategies for the Development of Maize Introgression Populations

**DOI:** 10.1371/journal.pone.0092429

**Published:** 2014-03-19

**Authors:** Eva Herzog, Karen Christin Falke, Thomas Presterl, Daniela Scheuermann, Milena Ouzunova, Matthias Frisch

**Affiliations:** 1 Institute of Agronomy and Plant Breeding II, Justus Liebig University, Giessen, Germany; 2 Institute for Evolution and Biodiversity, University of Münster, Münster, Germany; 3 KWS Saat AG, Einbeck, Germany; University of Guelph, Canada

## Abstract

Introgression libraries are valuable resources for QTL detection and breeding, but their development is costly and time-consuming. Selection strategies for the development of introgression populations with a limited number of individuals and high-throughput (HT) marker assays are required. The objectives of our simulation study were to design and compare selection strategies for the development of maize introgression populations of 100 lines with population sizes of 360–720 individuals per generation for different DH and 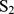 crossing schemes. Pre-selection for complete donor chromosomes or donor chromosome halves reduced the number of simultaneous backcross programs. The investigated crossing and selection schemes differed considerably with respect to their suitability to create introgression populations with clearly separated, evenly distributed target donor chromosome segments. DH crossing schemes were superior to 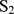 crossing schemes, mainly due to complete homozygosity, which greatly reduced the total number of disjunct genome segments in the introgression populations. The 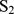 crossing schemes were more flexible with respect to selection and provided economic alternatives to DH crossing schemes. For the DH crossing schemes, increasing population sizes gradually over backcross generations was advantageous as it reduced the total number of required HT assays compared to constant population sizes. For the 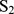 crossing schemes, large population sizes in the final backcross generation facilitated selection for the target segments in the final backcross generation and reduced fixation of large donor chromosome segments. The suggested crossing and selection schemes can help to make the genetic diversity of exotic germplasm available for enhancing the genetic variation of narrow-based breeding populations of crops.

## Introduction

Introgression libraries are valuable resources for the identification of alleles of agricultural interest in exotic germplasm. They facilitate the introduction of new genetic variation into elite breeding germplasm by providing favorable chromosome segments from wild or exotic species in an adapted genetic background [1], [2]. Ideally, an introgression library consists of a set of homozygous introgression lines (ILs) which carry short marker-defined chromosome segments from an exotic donor in a common genetic background. The concept was first described in tomato [3]. In the mean time, introgression libraries have been developed for the model species *Arabidopsis thaliana* [4], [5], and in many agriculturally important crops, such as rice [6], [7], barley [8], [9], wheat [10], [11], maize [12], [13] and rye [14].

Introgression libraries are usually developed by marker-assisted backcrossing followed by selfing or production of double haploid (DH) lines. The backcross process for their development is costly and labor-intensive if complete coverage of the donor genome by short evenly distributed target chromosome segments is to be achieved. Often additional backcross programs have to be run for the developed ILs in order to close gaps in donor genome coverage, or to shorten donor chromosome segments by additional recombination events [3], [9]. In spite of the high resource requirements, only incomplete donor genome coverage has been achieved for most of the reported introgression libraries [9], [14].

In previous simulation studies on introgression libraries, two generations of selfing were investigated for line development [15], [16]. Recent genetic studies in maize were based on ILs that underwent two to five generations of selfing [17]–[19]. The use of DH technology has to our knowledge not yet been investigated in simulation studies on the development of introgression libraries. However, *in vivo* induction of maternal haploids is currently a routine method of DH production in commercial maize breeding programs. The main advantage of the DH technology is that complete homozygosity can be obtained after only two generations. Inspite of this time-saving, the production of DH lines is still considerably more costly than conventional selfing [20]. Moreover, a current drawback of *in vivo* induction of maternal haploids in maize is that on average only one viable DH line can be derived from one backcross individual. It is therefore of economic interest to compare this method with 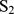 crossing schemes which require the same number of generations to evaluate the benefits of DH lines.

A possible approach to tackle the high costs required for the development of ideal introgression libraries would be to resort to introgression populations which are not perfect in appearance, but carry some additional donor segments outside the actual target segments. Such introgression populations could be developed with fewer individuals and marker assays. Complete coverage of the donor genome is desirable in order to capture the whole wealth of alleles of agricultural interest in the exotic donor. It is therefore one component of a minimum standard which introgression populations should meet. A second component are short, evenly distributed target donor chromosome segments in a clean adapted background, as they facilitate the use of the ILs in the following breeding process.

The design of the crossing scheme and the selection strategy are the most important factors that influence the distribution of donor chromosome segments in the introgression population. Falke et al. [16] suggested for the development of ideal introgression libraries that a chromosome-based selection strategy which pre-selects individuals carrying the donor alleles on complete chromosomes in generation 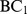 saves resources. Adapting and advancing this concept to crossing schemes with small population sizes might be an efficient approach to develop introgression populations with a limited number of marker assays.

The objectives of our simulation study were (1) to design selection strategies and crossing schemes for the development of maize introgression populations with limited resources, (2) to compare these selection strategies with respect to the distribution and length of donor chromosome segments and the required investments in terms of time, individuals and marker assays, (3) to give guidelines for the optimal experimental design for constructing introgression populations.

## Materials and Methods

### Software

All simulations were conducted in R version 3.0.0 [21] with the software package SelectionTools, which is available from http://www.uni-giessen.de/population-genetics/downloads.

### Genetic Model

A genetic model of maize with 10 equally sized chromosomes of 200 cM length was used for the simulations. Genetic markers for selection were equally spaced. The distance between two adjacent marker loci was 1 cM. All markers were polymorphic between donor and recipient. It was assumed that markers were analyzed with high-throughput (HT) assays. One HT assay comprised genotyping one individual at all marker loci in the linkage map. Recombination was modelled assuming no interference in crossover formation [22]. Each simulation of an introgression population of 100 ILs was replicated 1,000 times in order to reduce sampling effects and to obtain results with high numerical accuracy and a small standard error.

### Crossing Schemes

Four crossing schemes were investigated: 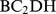, 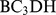, 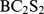, 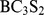. Each crossing scheme started with the cross of a homozygous donor and a homozygous recipient to create one 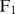 individual. The 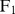 individual was backcrossed to the recipient to create a 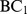 population of size 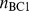. From the 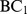 population, the best individuals with the highest values of selection indices for the respective selection strategy were selected. Each of the selected 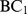 individuals was backcrossed to the recipient to create 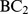 sub-populations of size 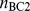. From these 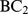 sub-populations, the best individuals with the highest values of the respective selection indices were selected. For the DH crossing schemes, *in vivo* induction of maternal haploids was assumed with a success rate of one viable DH line per backcross individual. For the 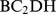 schemes, one DH line was thus created from each of the selected 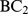 individuals. For the 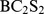 crossing schemes, the selected 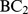 individuals were selfed to create a fixed number of 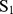 individuals. Each of the 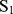 individuals was selfed again and one 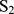 individual was created. For the 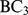 crossing schemes, each of the selected 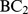 individuals was backcrossed to the recipient to create 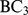 sub-populations of size 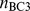. From these 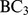 sub-populations, the best individuals with the highest values of the respective selection indices were selected. The generations 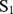, 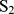 or DH of the 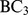 crossing schemes were carried out as described for the 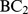 crossing schemes.

### Evaluation of Selection Candidates

The final introgression populations should consist of 100 ILs which guarantee an acceptable resolution of QTL detection in maize, and which can be immediately used in further breeding steps. Each IL should ideally carry a 20 cM chromosome segment from the donor to provide a complete and even coverage of the donor genome without overlap. The 20 cM chromosome segments are hereafter simply referred to as “target segments”. To determine the selection index for an individual with respect to a given target segment, we denote with 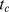 the donor genome proportion of the chromosome on which the target segment is located, with 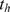 the donor genome proportion of the chromosome half on which the target segment is located and with 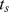 the donor genome proportion of the target segment itself. The values for the genetic background 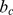, 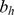, 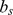 correspond to 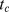, 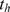, 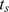 and denote the recipient genome proportion outside the respective chromosome region. Depending on the selection strategy, 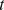 and 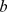 are used to define selection indices.

### Selection Strategies

We considered generations 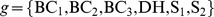 for selection. Generation DH was the generation in which homozygous diploid DH lines were available for selection. In each generation 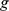, the genome was divided into selection regions that could either be 10 complete chromosomes, 20 chromosome halves or 100 target segments. For selection for complete donor chromosomes, a fixed number 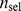 of best individuals for each of the chromosomes 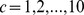 with the highest values for selection index 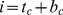 were selected. For selection for donor chromosome halves, a fixed number 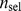 of best individuals for each of the chromosome halves 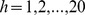 with the highest values for selection index 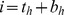 were selected. For selection for donor target segments, a fixed number 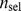 of best individuals for each of the target segments 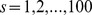 with the highest values for selection index 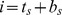 were selected.

Selection for complete donor chromosomes, donor chromosome halves and donor target segments were combined to form different selection strategies. Selection for complete donor chromosomes in a backcross generation is denoted by a C in the strategy name, selection for donor chromosome halves is denoted by an H, and selection for donor target segments is denoted by an S. For example, for strategy CH, selection for complete donor chromosomes was conducted in generation 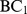 while selection for donor chromosome halves was conducted in generation 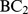. An overview of the investigated selection strategies is presented in Table 1. The investigated combinations of crossing scheme and selection strategy are listed in the first column of Table 2. For all selection strategies, the best 100 ILs for selection index 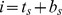 were selected in generation DH or 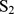, depending on the crossing scheme.

**Table 1:** Definition of the selection index 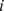 in generations 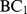, 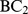, 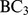, DH, 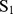, 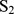 for different selection strategies for developing introgression populations.

	Generation				
Strategy	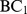	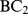	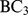	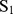	DH/ 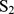
C	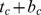	–	–	–	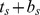
H	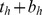	–	–	–	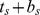
CC	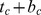	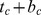	–	–	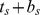
HH	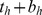	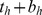	–	–	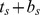
CH	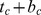	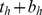	–	–	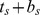
CCC	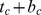	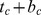	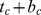	–	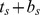
HHH	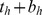	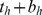	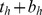	–	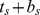
CHH	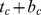	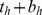	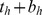	–	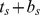
HHS	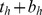	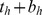	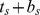	–	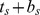

Selection for complete donor chromosomes (C), selection for donor chromosome halves (H) and selection for donor target segments (S) were combined to form different selection strategies (left column). 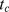, 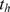 and 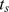 denote the donor genome proportions of the chromosome on which the target segment is located, of the chromosome half on which the target segment is located and of the target segment itself. 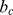, 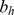 and 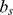 correspond to 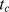, 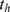, 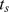 and denote the recipient genome proportion outside the respective chromosome region.

**Table 2:** Subdivision of the total population sizes 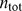 into sub-population sizes 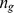 in generations 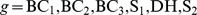 for different crossing and selection schemes for developing introgression populations.

	Generation				
Scheme	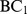	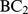	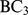	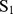	DH/ 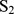
Basic crossing schemes					
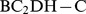	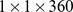	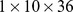	–	–	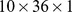
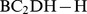	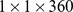	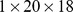	–	–	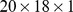
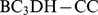	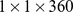	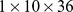	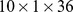	–	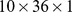
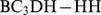	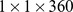	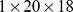	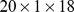	–	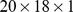
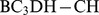	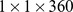	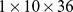	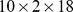	–	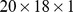
Crossing schemes with high selection intensity					
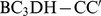	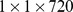	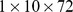	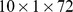	–	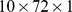
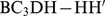	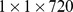	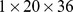	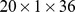	–	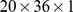
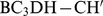	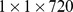	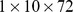	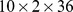	–	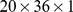
Crossing schemes with selection in the final BC generation					
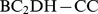	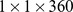	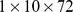	–	–	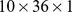
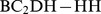	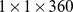	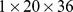	–	–	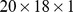
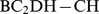	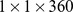	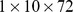	–	–	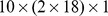
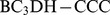	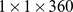	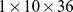	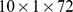	–	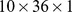
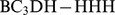	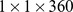	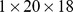	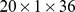	–	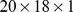
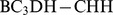	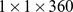	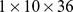	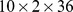	–	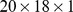
Crossing schemes with increasing population sizes					
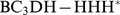	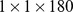	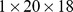	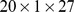	–	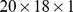
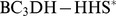	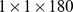	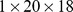	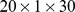	–	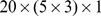
Basic crossing schemes					
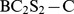	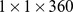	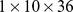	–	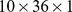	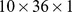
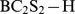	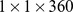	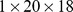	–	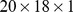	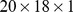
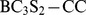	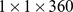	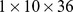	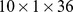	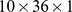	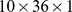
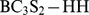	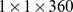	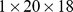	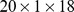	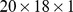	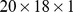
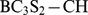	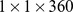	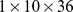	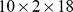	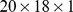	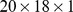
Crossing schemes with high selection intensity					
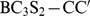	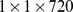	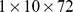	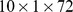	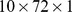	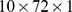
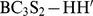	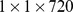	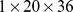	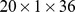	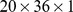	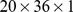
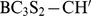	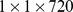	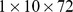	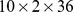	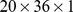	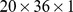
Crossing schemes with selection in the final BC generation					
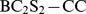	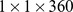	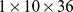	–	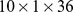	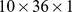
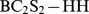	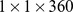	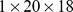	–	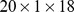	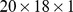
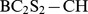	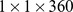	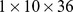	–	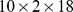	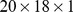
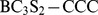	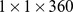	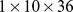	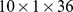	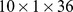	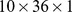
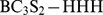	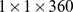	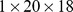	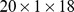	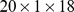	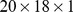
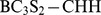	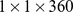	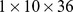	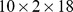	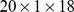	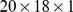
Crossing schemes with increasing population sizes					
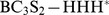	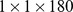	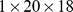	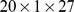	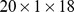	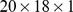
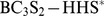	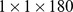	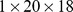	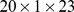	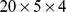	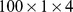

The total population size in generation 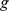 is defined as 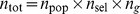. 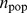: number of sub-populations in generation 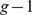; 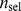: number of individuals selected from the sub-populations in generation 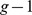; 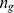: population size per sub-population in generation 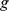.

### Population Sizes and Simulation Series

We investigated population sizes of 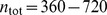 individuals per backcross generation. This should be within a range which can be realized in practical maize breeding programs. Variations in population size were investigated to determine both the effect on preserving the target segments up to line development as well as on recovering the genotype of the recipient outside the target segments.

In the first series of simulations, basic crossing schemes were investigated. Selection was carried out in generation 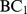 for basic crossing schemes with two backcross generations, and in generations 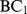 and 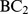 for basic crossing schemes with three backcross generations. The total population size per generation was kept constant at 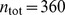 individuals in every generation 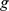.

In the second series of simulation, crossing schemes with high selection intensity were investigated. Population size was doubled compared to the basic crossing schemes 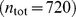 in every generation 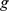, while the number of selected individuals was the same as for the basic crossing schemes. The crossing schemes with high selection intensity are denoted by 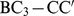, 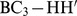 and 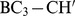 (Table 2). In the first and second series of simulations, all backcross individuals generated in the final backcross generation were used for line development for both DH and 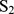 crossing schemes. One IL was derived from one backcross individual.

In the third series of simulations, crossing schemes with selection in the final backcross generation were investigated. 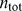 was doubled to 720 individuals in the final backcross generation for the DH crossing schemes 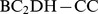, 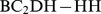, 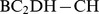, 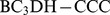, 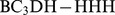, 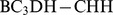. This increase in population size was necessary to enable selection and to keep 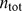 at 360 individuals in generation DH. For the corresponding 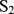 schemes, 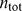 was kept at 360 individuals also in the final backcross generation.

**Figure 1:**
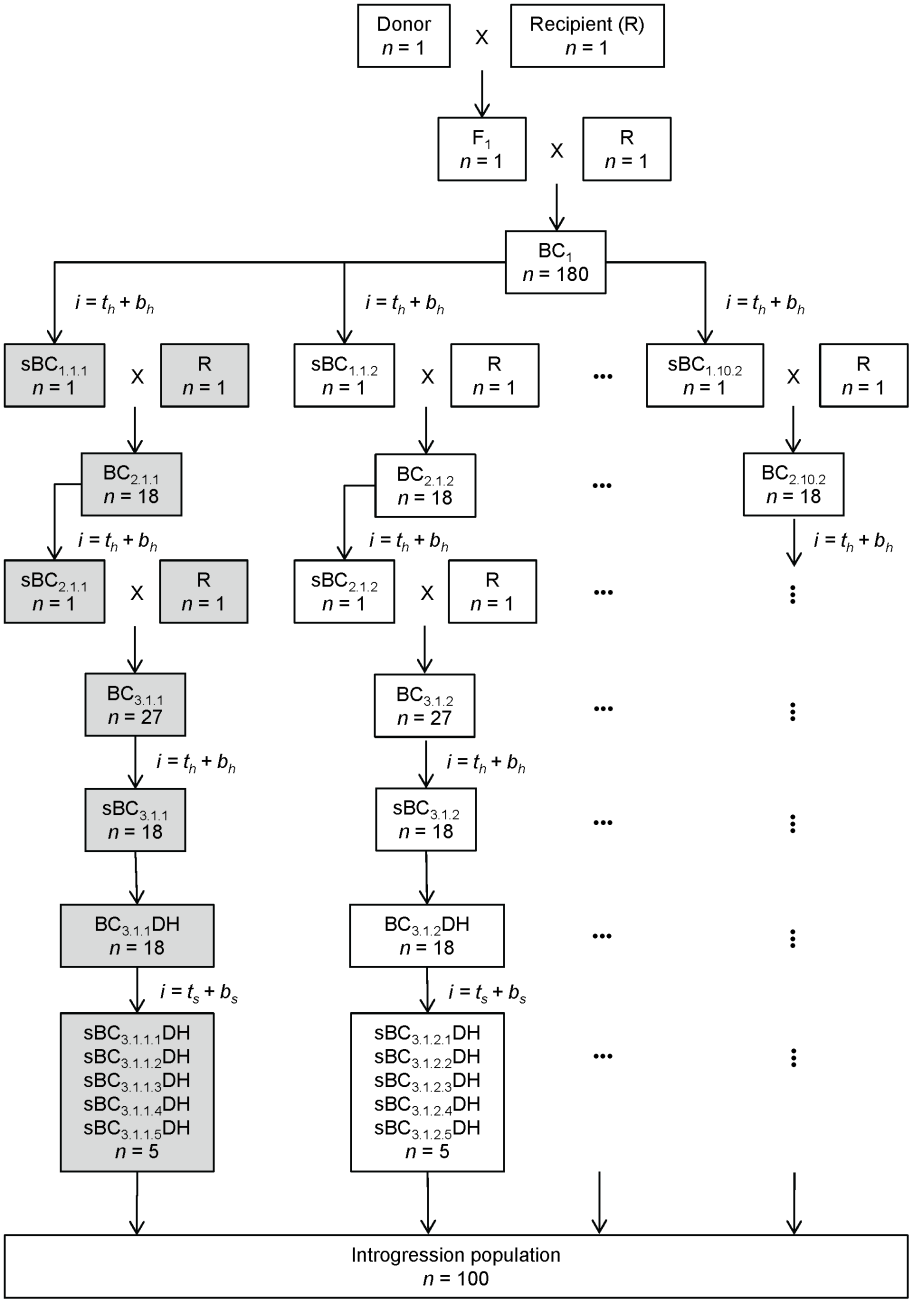
Schematic representation of crossing scheme 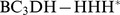. Crossing scheme 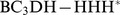 is characterized by increasing population sizes in the backcross generations and selection for donor chromosome halves in the final backcross generation. The parts highlighted in gray represent one branch of the crossing scheme. Sub-populations are indexed by 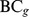, 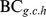 and 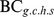, where 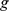 is the respective backcross generation, 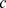 is the respective chromosome, 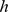 is the respective chromosome half, 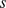 is the respective target segment; 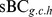 and 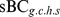 denote individuals selected for the respective selection regions.

**Figure 2:**
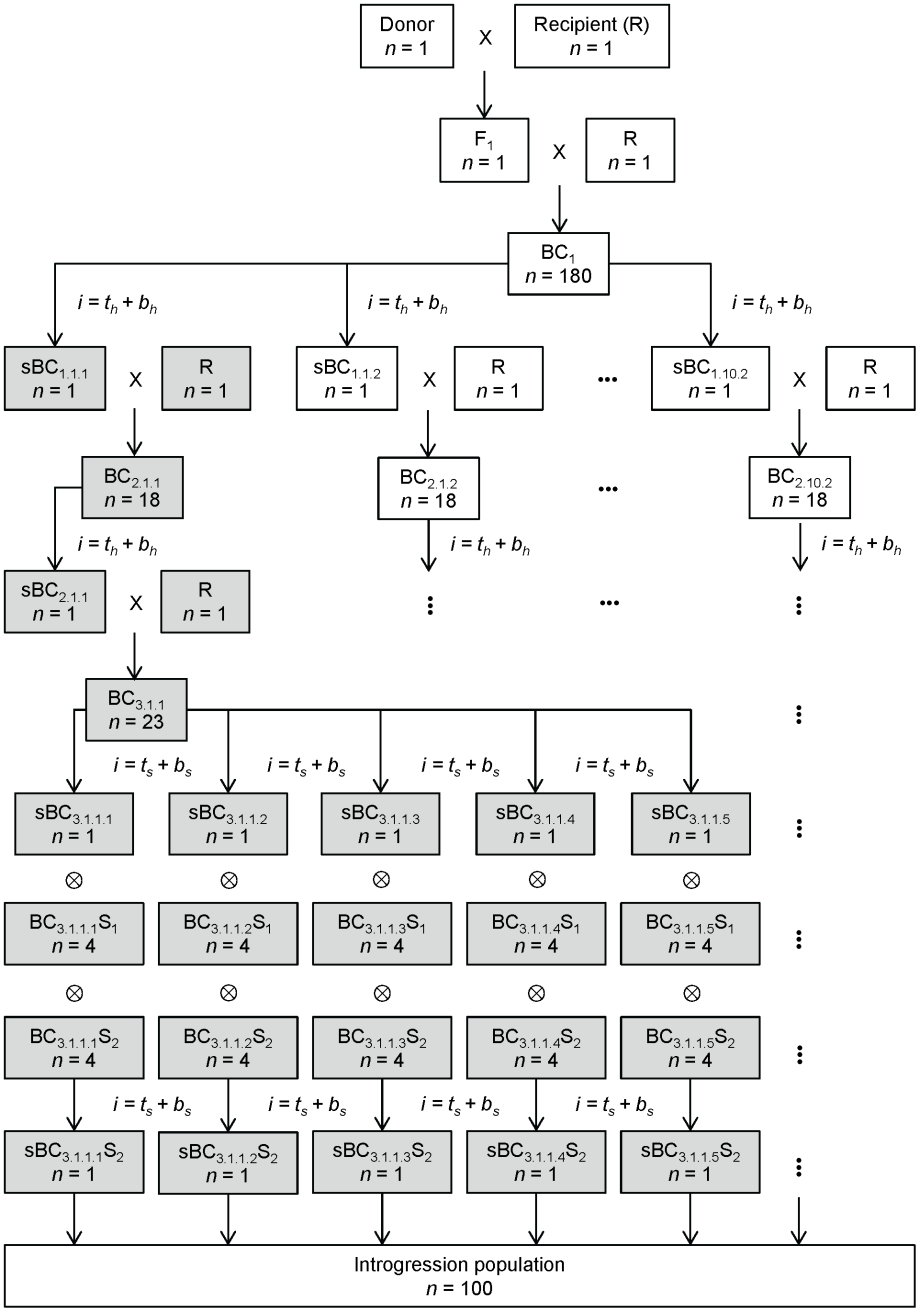
Schematic representation of crossing scheme 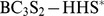. Crossing scheme 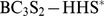 is characterized by increasing population sizes in the backcross generations and selection for target segments in the final backcross generation. The parts highlighted in gray represent one branch of the crossing scheme. Sub-populations are indexed by 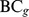, 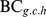 and 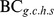, where 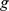 is the respective backcross generation, 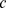 is the respective chromosome, 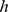 is the respective chromosome half, 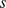 is the respective target segment; 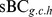 and 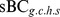 denote individuals selected for the respective selection regions.

In the fourth series of simulations, crossing schemes with increasing population sizes were investigated. Selection was conducted in the final backcross generation. The crossing schemes with increasing population sizes are denoted by 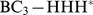 and 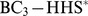. The details concerning the total population size 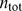 and population sizes in the sub-populations 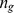 for all investigated combinations of crossing scheme and selection strategy are summarized in Table 2. Schematic representations of the crossing schemes 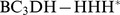 and 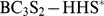 are given in Figure 1 and Figure 2 for illustration.

### Measures

To evaluate and compare introgression populations originating from different crossing and selection schemes, the following measures were determined: (a) the genome coverage of the donor 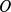 in percent, which is defined as the proportion of the donor genome which is covered by the introgression population, irrespective of whether by the target segments or other donor segments in the genetic background, (b) the depth of donor genome coverage 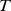, which is defined as the average number of ILs in which each donor allele appears in the introgression population, (c) the number of disjunct genome segments in the introgression population 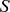, (d) the resolution of the introgression population 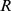 in cM, which is defined as the total genome length of the genetic model in cM divided by 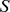, (e) the average number of donor segments per IL 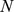, (f) the average length of donor segments per IL 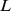 in cM, (g) the average total donor genome proportion of the introgression population 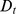 in percent, (h) the average donor genome proportion of the chromosomes carrying the respective target segments 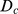 in percent, (i) the average donor genome proportion of the target segments 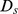 in percent.

## Results

High values for the donor genome coverage 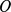 around 99% were observed for all crossing schemes (Table 3). However, the resulting introgression populations differed substantially in the values for the number of disjunct genome segments 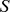, the total donor genome proportion 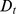, the donor genome proportion of the carrier chromosomes 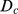 and the donor genome proportion of the target segments 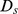. BC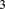 crossing schemes resulted in 2–3% lower values for 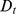 than 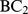 crossing schemes, even if the number of generations of selection was the same. For example, the basic crossing scheme 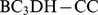 resulted in a 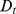 of only 5.0%, while crossing scheme 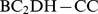 with selection in the final backcross generation resulted in a 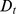 of 7.8%. An additional generation of selection in 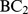 schemes only resulted in minor improvements of 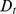 of 0.4–1.4% compared to the basic crossing schemes without selection. For example, scheme 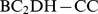 improved 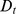 only by 0.5% compared to scheme 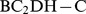.

**Table 3:** Measures evaluated for introgression populations resulting from different crossing and selection schemes.

Scheme	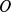	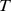	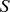	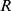	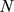	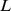	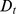	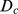	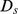	HT
Basic crossing schemes										
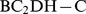	99.9	8.3	691	2.9	6.2	28.7	8.3	38.9	98.7	720
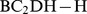	100.0	8.9	751	2.7	6.4	29.2	8.9	37.3	94.1	720
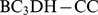	99.2	5.1	457	4.4	3.8	29.8	5.0	35.2	97.8	1080
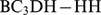	99.8	5.2	487	4.1	3.9	29.8	5.1	33.3	94.2	1080
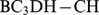	99.6	5.2	469	4.3	3.8	30.5	5.1	35.1	93.7	1080
Crossing schemes with high selection intensity										
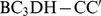	99.3	3.6	389	5.1	3.0	27.8	3.6	27.7	98.8	2160
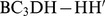	99.9	3.8	406	4.9	3.0	29.7	3.8	29.1	99.4	2160
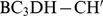	99.6	3.9	399	5.0	3.0	30.0	3.8	29.9	99.0	2160
Crossing schemes with selection in the final BC generation										
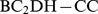	99.9	7.8	676	3.0	5.9	28.7	7.8	41.0	98.9	1440
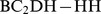	100.0	8.1	716	2.8	6.0	29.0	8.1	38.9	99.3	1440
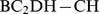	99.9	8.5	679	2.9	6.0	30.4	8.5	43.7	98.8	1440
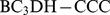	99.1	4.9	457	4.4	3.7	30.2	4.8	35.8	98.0	1800
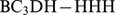	99.9	4.8	464	4.3	3.6	31.0	4.7	33.9	98.9	1800
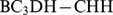	99.6	4.8	450	4.4	3.5	31.4	4.7	35.0	98.5	1800
Crossing schemes with increasing population sizes										
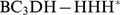	99.9	5.0	492	4.1	3.8	29.7	5.0	33.3	98.4	1440
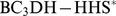	99.8	4.9	484	4.1	3.8	29.4	4.8	32.5	97.5	1440
Basic crossing schemes										
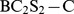	100.0	11.4	1021	2.0	7.3	26.4	9.3	41.4	97.6	720
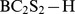	100.0	11.4	1073	1.9	7.4	25.9	9.3	36.3	90.4	720
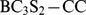	99.3	6.9	684	2.9	4.5	27.7	5.7	39.2	97.0	1080
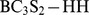	99.9	6.3	702	2.8	4.4	26.3	5.3	33.0	90.3	1080
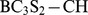	99.7	6.4	681	2.9	4.3	26.9	5.3	35.0	89.9	1080
Crossing schemes with high selection intensity										
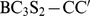	99.5	5.1	585	3.4	3.6	26.6	4.3	32.3	98.8	2160
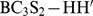	99.9	5.1	591	3.4	3.5	27.8	4.4	32.1	98.9	2160
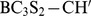	99.7	5.2	581	3.4	3.5	28.2	4.4	33.4	98.4	2160
Crossing schemes with selection in the final BC generation										
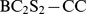	99.3	10.5	795	2.5	6.5	27.2	8.3	48.3	98.0	1080
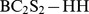	99.9	9.7	785	2.5	6.2	27.0	7.9	42.8	98.8	1080
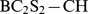	99.8	9.7	761	2.6	6.1	27.6	7.9	45.9	98.4	1080
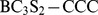	98.3	7.1	588	3.4	4.2	30.5	5.7	44.6	97.5	1440
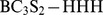	99.7	5.9	510	3.9	3.7	31.0	5.0	38.1	98.6	1440
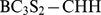	99.3	6.0	509	3.9	3.7	31.3	5.0	39.5	98.2	1440
Crossing schemes with increasing population sizes										
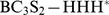	99.7	5.8	508	3.9	3.8	30.7	4.9	38.0	98.7	1440
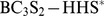	99.8	5.1	596	3.4	3.7	26.1	4.3	30.4	96.2	1400

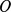
: donor genome coverage in percent; 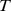: depth of donor genome coverage; 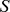: number of disjunct genome segments; 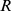: resolution; 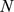: number of donor segments per IL; 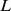: length of donor segments per IL in cM; 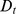: total donor genome proportion in percent; 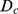: donor genome proportion of carrier chromosomes in percent; 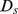: donor genome proportion of target segments in percent; HT: the required number of HT assays. Measures are arithmetic means over 1,000 replications.

**Figure 3:**
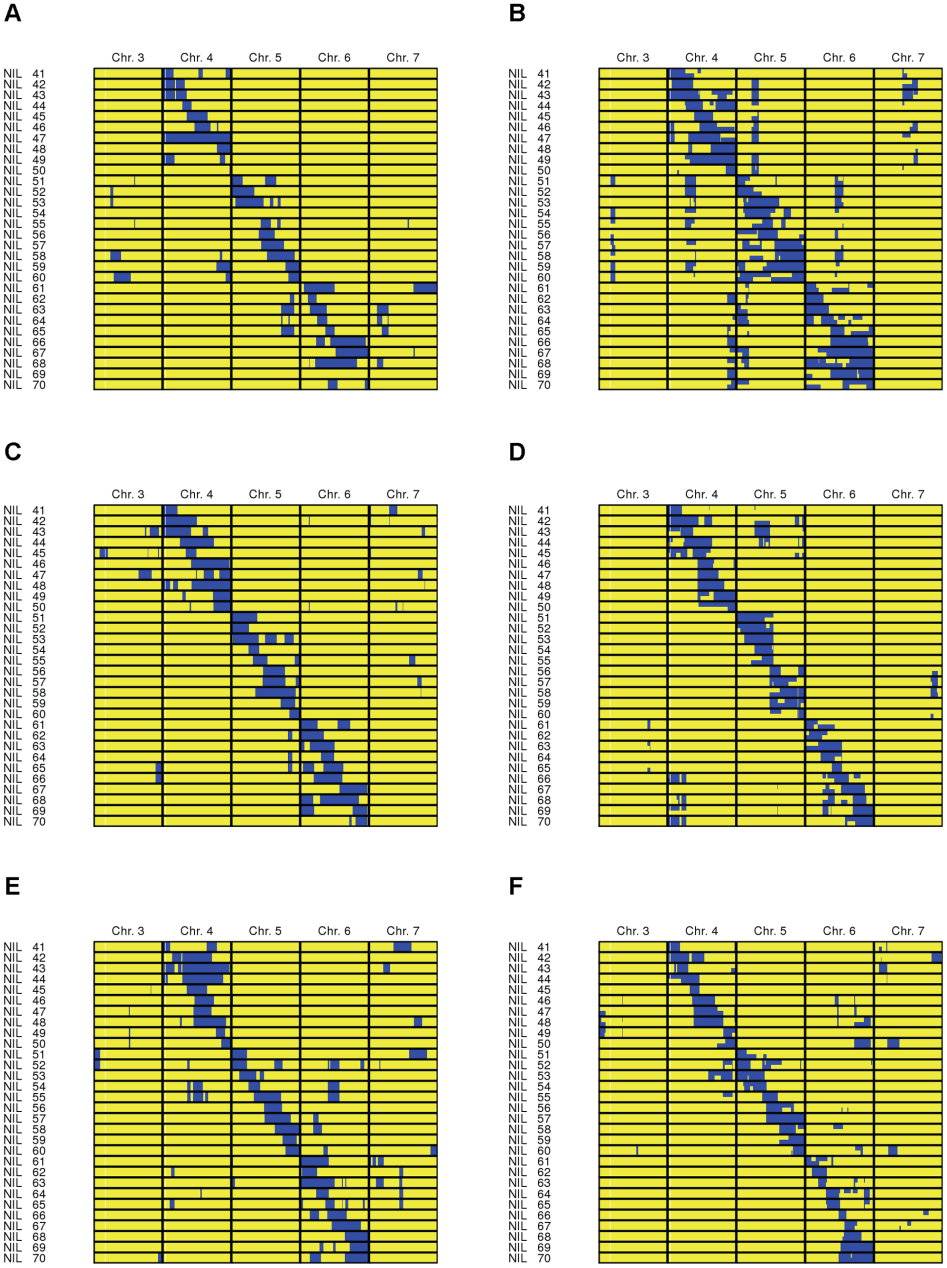
Graphical genotypes of introgression populations resulting from six different crossing schemes. A: 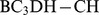; B: 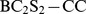; C: 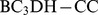; D: 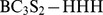; E: 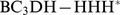; F: 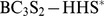. The graphical genotypes display the chromosomes 3 to 7 of ILs 41–70 and are examples from one simulation run. Chromosome segments which stem from the donor are displayed in blue, whereas chromosome segments which stem from the recipient are displayed in yellow. The graphical genotypes illustrate the differences between the alternative crossing schemes with respect to their suitability to create introgression populations with complete donor genome coverage and clearly separated, evenly distributed target donor chromosome segments.

The DH crossing schemes had in most cases better values for 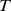, 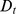, 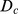, 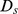 and especially 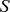 than the 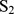 crossing schemes (Table 3). Very high values of 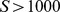 segments were observed for the basic crossing schemes 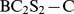 and 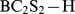. These crossing schemes had on average 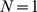 additional donor segment per IL compared to the corresponding DH crossing schemes. However, they were also characterized by incomplete homozygosity (Figure 3B). The 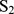 crossing schemes with selection in the final backcross generation required 360 individuals and HT assays less then the corresponding DH crossing schemes (Tables 2 and 3). Nevertheless, the differences between DH and 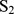 crossing schemes then diminished. For example, scheme 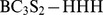 resulted in similar values for most measures as the corresponding scheme 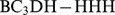 (Table 3).

**Figure 4:**
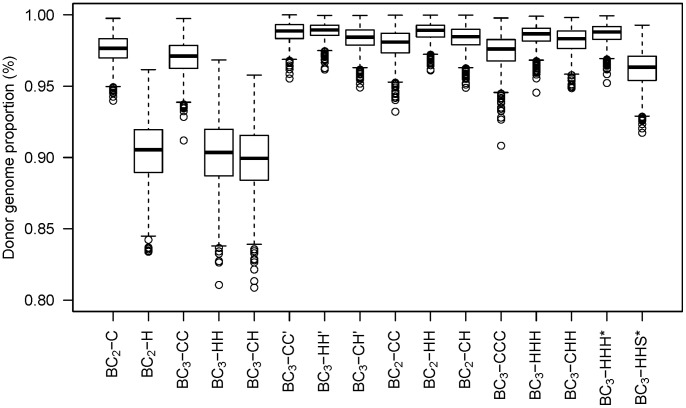
Donor genome proportion of target segments 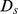 for all investigated 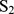 crossing schemes. The boxplots represent the distribution over 1,000 replications of the simulations. The basic crossing schemes 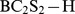, 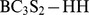 and 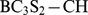 which select for donor chromosome halves are characterized by higher ranges for 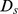.

The differences in the total donor genome proportion 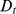 between selection for complete donor chromosomes and selection for donor chromosome halves ranged only between 0.1–0.7% for the same number of backcross generations and generations of selection. However, substantial differences were observed for the donor genome proportion of the carrier chromosomes 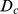 and the donor genome proportion of the target segments 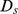. For selection for complete donor chromosomes, high values for 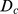 of up to 48% were observed. They were clearly visible in the graphical genotypes for schemes 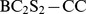 and 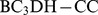 (Figure 3B and C). For selection for donor chromosome halves, the values for 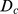 were much lower and did not exceed 42% (Table 3). Without selection in the final backcross generation, selection for donor chromosome halves resulted in substantially reduced values for 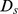. For example, the basic crossing schemes 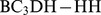 and 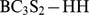 resulted in values for 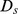 of only 94% and 90%. Moreover, the ranges for 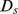 for these crossing schemes were substantially greater (Figure 4 for 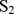 crossing schemes, for DH data not shown).

The basic crossing schemes 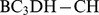 and 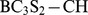 which combined selection for complete donor chromosomes and selection for donor chromosome halves resulted in similarly low values for 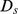 of 93.7% and 89.9% as selection for donor chromosome halves only (Table 3). In addition, the combined strategies CH and CHH resulted in high values for 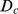 of up to 45.9%. The low values for 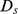 and the high values for 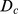 were reflected in the graphical genotype of scheme 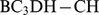, e.g. in ILs 47 and 54 (Figure 3A).

Doubling population sizes 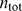 from 360 to 720 individuals in the crossing schemes with high selection intensity reduced the total donor genome proportion 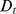 from 5.0–5.1% to 3.6–3.8% compared to the basic DH crossing schemes, and from 5.3–5.7% to 4.3–4.4% compared to the basic 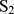 crossing schemes (Table 3). The donor genome proportion of the carrier chromosomes 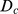 was reduced by about 4.2–7.5% for the DH crossing schemes, and by about 0.9–6.9% for the 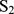 crossing schemes. The reduction of the donor genome proportion of the target segments 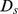 in combination with increased ranges that was observed with selection for donor chromosome halves in the basic crossing schemes was not observed in the crossing schemes with high selection intensity (Table 3 and Figure 4). 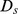 was increased by 5.2% for crossing scheme 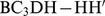 and by 8.6% for crossing scheme 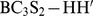 compared to the basic crossing schemes 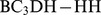 and 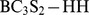. However, these improvements were only achieved with 2160 HT assays compared to 1080 HT asssays in the basic crossing schemes (Table 3).

The crossing schemes with selection in the final backcross generation resulted in values for 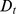 that were 1.1–1.2% higher for the DH crossing schemes und 0.6–1.4% higher for the 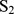 crossing schemes compared to the crossing schemes with high selection intensity. The ranges of 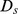 for selection for donor chromosome halves were about the same size as for the crossing schemes with high selection intensity (Figure 4). The average values for 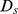 were 0.5% lower for scheme 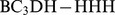 and 0.3% lower for scheme 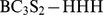 (Table 3). The number of required HT assays was reduced by 360 for the DH crossing schemes and by 720 for 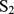 crossing schemes compared to the crossing schemes with high selection intensity. For the crossing schemes with selection in the final backcross generation, selection for donor chromosome halves was the most advantageous selection strategy with respect to the genetic background and to the target segments. Most notably, the crossing schemes 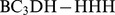 and 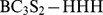 resulted in the lowest values for the donor genome proportion of the carrier chromosomes 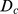. Compared to the most efficient basic crossing schemes 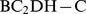 and 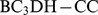, the crossing schemes 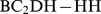 and 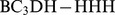 resulted in small improvements of both the genetic background and 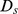. However, in both cases 720 additional HT assays had to be invested. For the 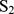 crossing schemes with selection in the final backcross generation, high values of 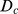 of 38.1–48.3% were observed. Large donor chromosome segments on the carrier chromosomes were also visible in the graphical genotypes for schemes 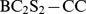 and 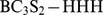 (Figure 3B and D). The high values for 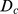 were associated with a considerable reduction of the number of disjunct genome segments 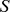 of 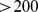 segments for the 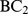 crossing schemes and of 100–200 segments for the 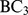 crossing schemes compared to the basic 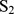 crossing schemes (Table 3).

The crossing schemes with increasing population sizes reduced the number of required HT assays for DH crossing schemes by 360 in comparison to the crossing schemes with selection in the final backcross generation and constant population sizes. The crossing schemes 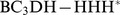 and 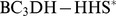 resulted in similar values for most measures as the crossing scheme 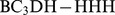. However, 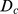 and 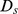 were slightly reduced for crossing scheme 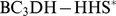. Compared to the most efficient basic crossing scheme 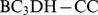, crossing scheme 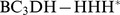 required 360 additional HT assays, but reduced 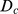 by 1.9% and increased 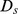 by 0.6%. The crossing scheme 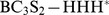 resulted with 38.0% in a much higher 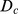 than the crossing scheme 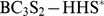 with 30.4%. For crossing scheme 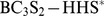, the average 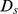 was only 96.2% and the range for 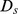 was higher than for the crossing schemes 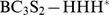 and 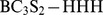 (Figure 4). However, 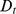 and 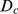 were the lowest for all investigated crossing schemes, with the exception of the crossing schemes with high selection intensity and 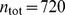 (Table 3). The clear-cut separation of the target segments is also visible in the graphical genotype (Figure 3F).

## Discussion

### Measures for Characterizing Introgression Populations

Measures for the description of introgression populations should allow to distinguish between introgression populations of different structure. Complete donor genome coverage 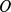 is desirable in order to make the complete genetic variation of the donor available for the breeding process. However, high values for 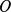 can also be caused by donor segments outside the target segments which could not be removed from the genetic background. 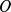 is therefore only informative if interpreted in relation to measures which reflect the distribution of the donor genome in the introgression population. A distinctive description of introgression populations is possible with the total donor genome proportion 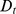, the donor genome proportion on the carrier chromosomes 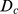 and the donor genome proportion of the actual target segments 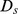.

A high total donor genome proportion 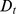 is often associated with a high number of disjunct genome segments 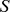. 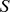 determines the resolution 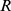, which is an important parameter for the accuracy of QTL detection. However, if 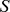 is greater than the number of ILs, the problem of overparameterization arises with classical linear model approaches. This issue has only in part been resolved by using statistical methods which pre-select a reduced number of ILs for the linear model [23].

High values for the donor genome proportion on the carrier chromosomes 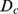 and the depth of donor genome coverage 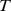 reflect undesired donor segments attached to the actual target segments. Such large donor segments which overlap between ILs have been reported to increase the risk of false-positive effects in QTL detection and reduce the power of QTL detection [24]. This is mainly a problem if linkage maps with large distances between adjacent markers of 10 cM or more are employed, because QTLs located between the last marker of the target segment and the next marker outside the target segment are incorrectly assigned to the target segments. With dense marker maps which are now available this problem should be overcome. However, large donor segments also increase the risk of linkage drag in the breeding process and often require further steps of separation [24].

Low values for the donor genome proportion of the target segments 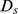 indicate a loss of target segments and potentially useful alleles. This is a problem that arises with small population sizes as were investigated in the present study [16]. Even if the missing target segments are present in the genetic background of other ILs, this might impair QTL detection and the further use of the ILs for the breeding progress.

We therefore argue that short non-overlapping target segments in a clean recipient background are advantageous also with dense marker maps. For 20 cM target segments and a genomic model of 10 equally sized chromosomes of 200 cM length, this corresponds to 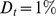, 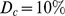 and 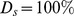 in the ideal case. The effort and time required for developing introgression populations with such characteristics is beyond the scope of most breeding programs. With the limited population sizes and number of HT assays investigated in this study, these ideal values could not be achieved with two or three backcross generations (Table 3). We therefore considered those crossing and selection schemes as efficient which with a given limited resource input resulted in the highest coverage of target segments 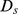 in combination with low overlap of target segments reflected in 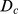 and 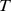 and a low total donor genome proportion 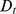.

With respect to QTL detection, it can be expected that the optimal values for the suggested measures will depend on the statistical method and the genetic architecture of the trait. They could be determined for a given statistical method by including QTLs of different number and effect in future simulation studies. We plan further investigations in this area of research.

### Crossing Schemes


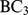
 crossing schemes had 2–3% lower values for the total donor genome proportion 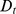 than 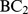 crossing schemes (Table 3), even if no selection for the genetic background was conducted in generation 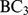. Selection in generation 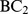, as was investigated with the crossing schemes 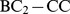, 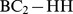 and 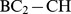, only resulted in a reduction of 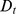 of 0.4–1.4% compared to the basic crossing schemes 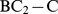 and 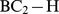 (Table 3). An explanation for this comparatively small reduction is that the limiting factor for the reduction of 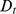 is the number of recombinations during meiosis. Hence, even though 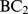 crossing schemes have a time advantage, the effect of a third backcross generation cannot be compensated by investing in additional marker analyses. We therefore conclude that 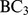 crossing schemes result in introgression populations with an improved structure, and that the time investment in the additional backcross generation is worthwhile.

DH crossing schemes were for most measures superior to the corresponding 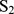 crossing schemes. The differences were most pronounced in the number of disjunct genome segments 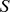. Even though the 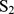 schemes on average had a slightly higher number of donor segments per IL 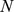, it seems that the very high values for 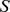 that were observed especially in the 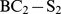 crossing schemes mainly had to be attributed to incomplete homozygosity (Figure 3B). It can be expected that introgression populations with 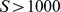 segments in 100 ILs (Table 3) are not suitable for effective QTL detection. We therefore conclude that the DH method is essential for short crossing schemes with only two backcross generations.

A drawback of the DH method is that with current protocols of *in vivo* DH induction of maternal haploids, only a very limited number of viable DH lines can be derived from one backcross individual. We expect that our assumption of one DH line per backcross individual is a conservative, but realistic estimate. In contrast, with selfing, many progenies can be derived from one selected backcross individual. In the 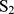 crossing schemes, it is consequently comparatively cheap and easy to conduct selection in the final backcross generation. For the DH schemes, selection in the final backcross generation could only be conducted if population size in this generation was higher than the desired number of final DH lines. As a result, the 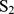 crossing schemes with selection in the final backcross generation required 360 HT assays less than the corresponding DH schemes (Table 3). Moreover, the selected fractions of best backcross individuals were much greater for the DH than for the 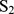 crossing schemes (Table 2). This resulted in a lower selection intensity for both the selection region of the final backcross generation and the genetic background in the DH crossing schemes. We therefore suggest that a comparison of DH and 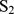 crossing schemes should take the distinctive features of both methods into account. The evaluation of efficiency should also be based on the number of required HT assays. Considering this, 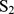 crossing schemes which exploit their selection advantages represent economic and easy-to-handle alternatives to DH crossing schemes.

### Selection Strategies for Small and Constant Population Sizes

For a given genetic model and crossing scheme, the selection strategy is the most important factor that influences the structure of the resulting introgression population. In the following paragraphs, different aspects such as the length of the selection regions, the number of generations of selection and the required population sizes for effective selection are discussed.

Selection strategies which pre-select individuals carrying complete donor chromosomes reduce the number of simultaneous backcross programs to the number of chromosomes [16]. They are therefore suitable for breeding programs with limited resources. However, for long chromosomes of 200 cM length, selection for complete donor chromosomes preserved large donor chromosome segments on the carrier chromosomes up to line development (Figure 3C and B). This was reflected in high values for the proportion of donor genome on the carrier chromosomes 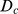 of up to 48% (Table 3). The selection regions for selection in the backcross generations were therefore reduced to donor chromosome halves for selection strategies H, HH and HHH. In all four series of simulations, selection for donor chromosome halves resulted in the desired reduction of 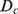 compared to selection for complete donor chromosomes (Table 3). Other measures for the genetic background were approximately equivalent. We therefore conclude that for crop species with long chromosomes such as maize, wheat or rapeseed, selection for donor chromosome halves reduces the length of the donor segments attached to the actual target segments and the risk of linkage drag.

However, for crossing schemes without selection in the final backcross generation and constant population sizes of 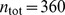 individuals, selection for donor chromosome halves resulted in a considerable reduction of the donor genome proportion of the target segments 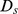 of up to 7%. Moreover, the estimated values for 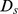 were less reliable for these crossing schemes, *e.g.*, in schemes 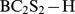 and 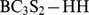 (Figure 4). These findings have to be attributed to the small population sizes 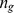 in the sub-populations and the structure of the selection index 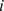. In generation DH or 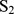, population sizes were reduced to 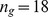 individuals with selection for donor chromosome halves (Table 2). Without selection in the final backcross generation, around 50% of the ILs developed from the backcross individuals are expected to carry no donor allele at a given locus within the respective target segment. The probability to find five ILs with complete donor target segments for the introgression population was therefore even further reduced. As the selection index 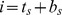 weighed the target segments and the genetic background equally, a clean genetic background sometimes outweighed a reduced 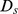 and led to the observed loss of target segments in these small sub-populations. We therefore conclude that a sufficiently large population size is the crucial factor for the successful application of selection for donor chromosome halves.

A loss of target segments caused by small population sizes was also observed for the basic combined selection strategy CH which selected for complete donor chromosomes in generation 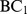 and for donor chromosome halves in generation 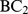. In addition, the combined strategies CH and CHH resulted in high values for the donor genome proportion on the carrier chromosomes 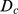 of up to 45% (Table 3). This can be explained by the efficient selection for complete donor chromosomes from the comparatively large 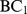 population of 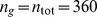 individuals (Table 2). The pre-selected complete donor chromosomes are in large part preserved up to line development. The combination of missing target segments with large donor chromosome segments on carrier chromosomes was also reflected in the graphical genotype for scheme 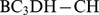, *e.g.*, in IL 47 and 54 (Figure 3A). The selection strategies CH and CHH therefore combine the drawbacks of both selection for complete donor chromosomes and selection for chromosome halves. They are not suitable for crossing schemes with small and constant population sizes, in which the population sizes 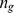 in the sub-populations are subsequently reduced over the backcross generations. We conclude that for for small breeding programs with a constant population size of 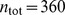 and a limited number of HT assays for selection, selection strategies which only select for complete donor chromosomes in the backcross generations should be employed in both DH and 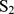 crossing schemes to avoid the loss of target segments.

### Finding more Carriers of Donor Target Segments for Line Development

To employ selection for donor chromosome halves effectively for reducing the donor genome proportion of the carrier chromosomes 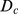 without losing the target segments, it is necessary to increase the frequency of carriers of donor target segments for line development. Using larger population sizes is a straightforward solution for this problem, which in addition can improve the overall structure of introgression populations. The crossing schemes with high selection intensity and double population sizes of 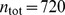 individuals resulted in small improvements of the total donor genome proportion 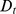 of about 1–1.5% compared to the basic crossing schemes (Table 3). The desired increase in the donor proportion of the target segments 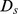 was achieved. For selection for donor chromosome halves, 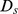 was increased by 5.2–8.6%. Selection for donor chromosome halves was then even superior to selection for complete donor chromosomes. Moreover, the donor genome proportion on the carrier chromosomes 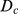 was reduced by up to 7.5%, indicating an improved separation of target segments. The observed improvements were greater for the DH than for the 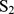 crossing schemes. Nevertheless, the comparatively small improvements of the introgression populations required 1080 additional HT assays. We therefore conclude that such large population sizes are only suitable for breeding programs with access to DH technology, less stringent resource restrictions and high requirements with respect to the genetic background. If the requirements concerning the structure of the introgression population are not that high, it might be more economic to increase population size only in the final backcross generation and/or to invest in additional HT assays only in this generation.

For crossing schemes with selection in the final backcross generation, the total donor genome proportion 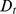 was similar to the values of the basic crossing schemes, and about 1% higher than for the crossing schemes with higher selection intensity (Table 3). However, the average values for the donor genome proportion of the target segments 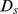 were similar to the crossing schemes with higher selection intensity (Table 3) and the ranges were effectively reduced (Figure 4). Moreover, the number of required HT assays was reduced by 360 for the DH crossing schemes and by 720 for 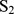 crossing schemes compared to the crossing schemes with higher selection intensity (Table 3). The decision for doubling population sizes requires the same resources as would be required for generating an additional introgression population. This large effort seems not to be justified by the relatively small improvements compared to the basic crossing schemes. We therefore conclude that selection in the final backcross generation is the more efficient solution for both DH and 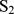 crossing schemes.

Selection for donor chromosome halves was the best strategy with selection in the final backcross generation for both DH and 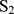 crossing schemes (Table 3). However, for the DH schemes, only small improvements for schemes 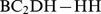 and 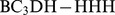 were observed compared to the most efficient basic crossing schemes 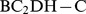 and 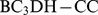 (Table 3). For these small improvements, 720 additional individuals and HT assays had to be invested. For the 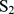 schemes, considerable reductions in 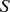 of 174 and 236 segments were observed for schemes 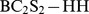 and 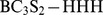 with selection in the final backcross generation compared to the basic crossing schemes 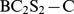 and 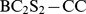. 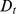 was only slightly reduced. However, the donor genome proportion on carrier chromosomes 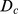 was in general very high for the crossing schemes with selection in the final backcross generation with 38–48%. This indicates a fixation of the selection regions of the final backcross generation (Figure 3B and D). In schemes 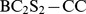 and 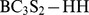, complete donor chromosomes and donor chromosome halves still appear as blocks around the target segments. These blocks lead to an overlap of donor segments between ILs that reduces the effective resolution of the introgression population for QTL detection. The overlap also hampers the further use of the ILs in the breeding process, as further steps of separation of the target segments by backcrossing are required. We therefore conclude that the crossing schemes with selection in the final backcross generation have the potential to improve the resulting introgression populations at moderate cost. However, for the DH crossing schemes, the number of required HT assays and individuals has to be reduced. For the 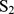 crossing schemes, the fixation of large donor chromosome segments has to be avoided. Optimizations of the respective crossing schemes are presented in the following.

### Increasing Population Sizes Over Backcross Generations

With constant population sizes of 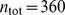 individuals, the population size in generation 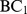 was large in relation to the genetic gains that could be achieved by selecting a comparatively small fraction of 10 or 20 individuals (Table 2). Starting with smaller population sizes in generation 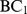 and gradually increasing population sizes in the following backcross generations was therefore an efficient option to reduce the overall number of required individuals and HT assays for selection in the final backcross generation. Larger population sizes in generation 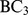 also enabled selection for target segments, which was investigated as an option to avoid the fixation of large donor chromosome segments especially for the 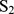 crossing schemes.

The schemes 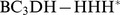 and 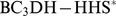 resulted in similar values for all measures (Table 3). However, 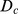 and 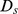 were slightly lower for scheme 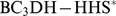. We therefore conclude that selection for target segments already in the final backcross generation is not efficient for DH crossing schemes. In comparison to the best but also very expensive scheme 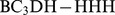 with selection in the final backcross generation, scheme 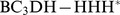 can be considered equivalent, but required 360 individuals and HT assays less. In comparison to the more economic basic crossing scheme 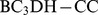, scheme 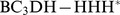 improved 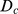 and 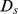 and thus the separation of target segments. This is also visible in the graphical genotype (Figure 3). The investment in the additional 360 HT assays seems therefore worthwhile (Table 3).

The scheme 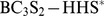 resulted in better values than the schemes 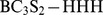 and 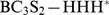. Most notably, it resulted in a much lower 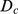 of 30% compared to 38%. Scheme 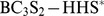 resulted in a 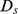 that was 2.4% lower compared to schemes 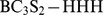 and 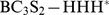 and the ranges for 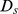 were higher (Figure 4). Nevertheless, it resulted in the lowest values of 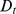 and 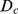 and the best separation of target genes of all investigated DH and 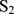 crossing schemes with comparable population sizes. The comparatively high value of 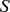 of 596 segments in combination with reduced values for 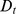 can in this case be explained by a greatly improved separation of target segments compared to the other 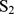 schemes with selection in the final backcross generation. The improved separation of target segments is also visible in the graphical genotype (Figure 3F). This was achieved with 40 HT assays less (Table 3). We therefore expect that scheme 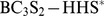 will result in an improved power of QTL detection, and recommend selection for target segments in the final backcross generation for 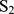 crossing schemes.

Compared to the best but expensive comparable DH crossing scheme 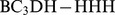, the 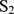 crossing scheme 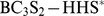 resulted in similar values and required 400 HT assays less. Overall, we conclude that increasing population sizes over backcross are advantageous and economic for both DH and 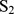 crossing schemes. Moreover, crossing scheme 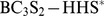 can provide a cheap alternative to comparable DH crossing schemes.

### Conclusions

Our study has shown that introgression populations with complete coverage of the donor genome and reasonably clean recipient background can be developed with a limited number of backcross individuals and HT assays. It has provided further insight on how different crossing and selection schemes influence the structure of the resulting introgression populations. The guidelines which have been derived for maize are transferable to other crop species with similar number and length of chromosomes. For crops with different genome size, some considerations are discussed in the following.

Rapeseed is a crop with a large genome of 19 chromosomes, for which efficient protocols of microspore culture are available for DH production. For the large genome of rapeseed, it can be expected that the values for the total donor genome proportion 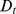 will be lower than those observed for the smaller genome of maize. With the investigated selection index 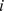, the selection pressure on the carrier chromosomes will be reduced with increasing genome size and number of chromosomes. It might therefore be an interesting option for rapeseed to put more weight on the background markers on the carrier chromosomes to achieve an efficient reduction of 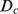. As with microspore culture many DH lines can usually be derived from one backcross individual, the advantages of DH production should be more pronounced than for maize. However, the optimal selection strategies for DH crossing schemes in rapeseed should then be similar to those for selfing in maize.

Sugar beet is a crop with a small genome of 9 chromosomes, for which the guidelines for selfing should be most relevant. In smaller genomes, equivalent values of 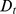 can usually be reached with smaller population sizes and with fewer backcrosses. However, the average length of the chromosomes in cM is also much shorter than in maize. This implies that fewer crossovers occur per meiosis, and that it might require more individuals and backcross generations to effectively separate the target segments. The combined effects of genome size and chromosome length will also depend on the desired number and length of the target segments.

Simulations can considerably facilitate the planning process for the development of introgression populations in different crop species. The derived guidelines can help breeders and geneticists to enhance the genetic variation of narrow based breeding populations of crops.
